# The influence of different glucose tolerance on QTc interval: a population-based study

**DOI:** 10.1186/s12872-023-03081-6

**Published:** 2023-01-25

**Authors:** Ning Lin, Hongmei Zhang, Xiaoyong Li, Yixin Niu, Hongxia Gu, Shuai Lu, Zhen Yang, Qing Su, Li Qin

**Affiliations:** 1 Department of Endocrinology, Chongming Hospital affiliated to Shanghai University of Health & Medicine Science, Shanghai, China; 2 Department of Endocrinology, Xin Hua Hospital Affiliated to Shanghai Jiao Tong University School of Medicine, Shanghai, China; 3 Department of Endocrinology, Shenzhen Hospital, Southern Medical University, Shenzhen, China

**Keywords:** QTc interval, Abnormal glucose metabolism, Type 2 diabetes

## Abstract

**Background:**

Corrected QT (QTc) interval has been reported to be associated with type 2 diabetes. This study aimed to explore the relationship between different glucose tolerance and QTc intervals among middle-aged and older Chinese individuals.

**Methods:**

We conducted a cross-sectional analysis that included 9898 subjects (3194 men and 6704 women) in a Chinese population. Glucose tolerance was studied during the oral glucose tolerance test (OGTT). Insulin, blood pressure, hemoglobin A1c (HbA1c), serum lipids, hepatic transaminases and waist-to-hip ratio were assessed. The QTc interval was derived from ECG recordings, and the subjects were stratified based on different glucose tolerance.

**Results:**

QTc interval levels were increased significantly in the subjects with abnormal glucose metabolism compared with the normal glucose regulation group. Multiple regression analyses showed that the QTc interval was significantly associated with fasting plasma glucose, 2-h OGTT plasma glucose and HbA1c. The odds ratio of prolonged QTc was 1.396 for impaired glucose regulation (IFG)/impaired fasting glucose (IGT) (95% CI 0.126–1.730), and 1.342 for type 2 diabetes (95% CI 0.142–1.577) after all potential confounders were adjusted.

**Conclusions:**

Impaired glucose tolerance (IGR) and diabetes are associated with prolonged QTc intervals among middle-aged and older Chinese individuals. Abnormal glucose regulation can be used to monitor the QTc interval in the population.

## Introduction

The risk of sudden death is increased in patients with diabetes mellitus and impaired glucose tolerance [1, 2]. Abnormal cardiac autonomic nervous system function is one of the most significant complications of diabetes [3]. The QT interval is defined as the total time required for ventricular myocardial depolarization and repolarization [4]. Corrected QT (QTc) is very important for the prediction, risk assessment and treatment of ventricular arrhythmias [2]. The relationship between a prolonged QTc interval and an increased risk of sudden death has been traditionally explored in familial long QT syndrome sudden cot death, and congestive heart failure, as well as in adults with diabetes mellitus [2, 5–8].

Although several studied have described the relationship between QTc interval prolongation, diabetic complications, and an increased mortality rate in adults [5, 9–11], the influence of abnormal glucose metabolism on the QTc interval has not been fully clarified. Previous studies have confirmed that the prevalence of prolonged QTc interval is increased in people with type 1 and type 2 diabetes as compared to non-diabetics, and the abnormalities in cardiac repolarisation may be caused by complications of diabetes rather than diabetes itself [5, 10, 12, 13]. Most recently, Kurnaz E et al. found that QTc prolongation already exists in a significant proportion of children and adolescents with newly diagnosed type 1 diabetes [14].

However, up to date, only a few studies have reported conflicting data on the relationship between the QTc interval and glucose metabolism regulation, especially the status of impaired glucose regulation (IGR) [5, 6, 10, 12]. In this study, we aimed to investigate the influence of different glucose tolerance on QTc intervals in a cross-sectional study of Chinese individuals aged 40–70 years.

## Material and methods

### Study population

The present study is one part of the risk evaluation of cancers in Chinese diabetic individuals, i.e., the longitudinal study (REACTION) which was a population-based cross-sectional study among middle-aged and elderly Chinese individuals in 25 communities across mainland China. The studied individuals were aged 40 to 70 years old. The details of the study design have been described previously [15, 16]. All participants came from the Chongming District in Shanghai, China and 9930 suitable subjects were recruited. After the exclusion of 1940 individuals with self-reported coronary heart disease, or taking medicine affecting heart rhythm, 7990 subjects (2489 men and 5501 women) were found to be suitable for the current analysis. Written informed consent was obtained from all of the participants. Approval was given by the Institutional Review Board of Xinhua Hospital affiliated with Shanghai Jiaotong University School of Medicine.

### Data collection

A standardized questionnaire was used by trained physicians to collect the baseline data. The measurements of weight, height, waist circumference, hip circumference and blood pressure have been described previously. Smoking (yes/no) and alcohol drinking (yes/no) were estimated with an interview preceding the physical examination. Body mass index (BMI) was calculated as weight in kilograms divided by the square of height in metres.

A 12-lead ECG was recorded at a paper speed of 50 mm/s on a six-channel recorder. The QT interval was measured from the beginning of the QRS complex to the end of the T-wave. The QT interval corrected for the previous cardiac R-R cycle length (QTc) was calculated according to the formula proposed previously and known as Bazett’s formula: QTc = QT/(RR) 1/2 [17]. QTc is the mean of QTc from five consecutive cycles in lead V5. QTc > 440 ms was considered abnormally prolonged [18].

After overnight fasting for at least 10 h, fasting and 2-h oral glucose tolerance test (OGTT) blood samples were collected from all the participants in tubes containing EDTA centrifuged at 4 °C and stored at − 80 °C untill analysis. Fasting glucose, 2-h OGTT plasma glucose, triglycerides, total cholesterol (TC), high-density lipoprotein (HDL) cholesterol, low-density lipoprotein (LDL) cholesterol, and serum creatinine (Scr) were measured on an automatic analyser (Hitachi 7080; Tokyo, Japan). The homeostasis model assessment of insulin resistance (HOMA-IR) was calculated based on the equation described by Matthews et al. [19].

### Definitions of impaired glucose regulation and type 2 diabetes

Impaired glucose regulation was defined as impaired fasting glucose (IFG, fasting plasma glucose level ≥ 6.1 and < 7.0 mmol/l) and/or impaired glucose tolerance (IGT, 2-h OGTT plasma glucose level ≥ 7.8 and < 11.1 mmol/l). Isolated IFG: fasting plasma glucose ≥ 6.1 mmol/l and < 7.0 mmol/l and a 2 h OGTT plasma glucose < 7.8 mmol/l. Isolated IGT: 2-h OGTT plasma glucose ≥ 7.8 mmol/l and < 11.1 mmol/l and a fasting glucose < 6.1 mmol/l. IFG/IGT: fasting plasma glucose between 6.1 and 6.9 mmol/l and a 2-h OGTT plasma glucose 7.8–11.0 mmol/l. Type 2 diabetes was diagnosed by the 1999 World Health Organization criteria (fasting plasma glucose level ≥ 7.0 mmol/l and/or a 2-h OGTT plasma glucose level ≥ 11.1 mmol/l [20]. A fasting glucose level < 6.1 mmol/l and a 2-h OGTT plasma glucose level < 7.8 mmol/l were defined as normal glucose regulation (NGR).

### Statistical analysis

Data management and statistical analysis were performed with the SPSS Statistical Package (version 22.0; SPSS Inc., Chicago, IL). Normally distributed data were expressed as means ± SD, whereas variables with a skewed distribution were reported as median (inter-quartile range). Comparisons of means and proportions were performed with the *t*-test and χ^2^ tests, respectively. Crude and partial correlation and multivariable stepwise regression analysis were used to investigate the association of QTc interval with cardiovascular and metabolic related parameters. A multiple linear regression analysis was performed to determine the associations of different glucose tolerance with QTc interval. Finally, a multivariate logistic regression model was used to evaluate the ORs and 95% CIs of different glucose tolerance for prolonged QTc. The statistical analyses were adjusted for potential confounders including age, sex, insulin, SBP, DBP, CHOL, TG, smoking, and drinking. *P* < 0.05 was considered statistically significant.

## Results

### Clinical characteristics of the participants

**Fig. 1:**
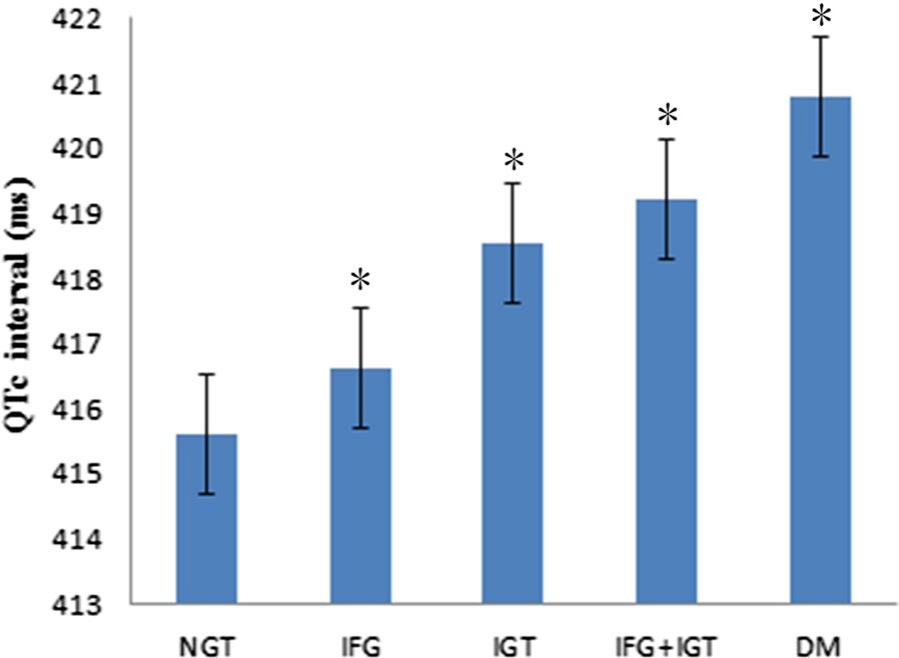
Adjusted means (± SD) of QTc interval in subjects with NGR, IGR (isolated IFG, isolated IGT), combined IFG/IGT and diabetes mellitus (DM). Subjects with type 2 diabetes mellitus and IGR (isolated IFG, isolated IGT and IFG/IGT) had higher level of QTc interval than those with NGR (both *p* < 0.0001). There was no significant difference among the subgroups of IGR (*p* = 0.35) and between the IGR and type 2 diabetes mellitus groups (*p* = 0.61)

The clinical characteristics of the participants stratified by glucose tolerance are summarized in Table 1. The study involved 3412 participants with NGR, 2475 with IGR and 1833 with type 2 diabetes. Among the participants with IGR, 773 (9.67%) had isolated IFG, 1255 (15.7%) had isolated IGT, and 717 (8.97%) had combined IFG/IGT. The NGR and diabetes groups had the most favorable and unfavorable metabolic profiles, respectively. Across different glucose tolerance levels, the subjects with higher blood glucose levels were more likely to be older, and have a higher waist-to-hip ratio, higher lipid profiles, and higher levels of blood pressure. Some liver and kidney function indicators (such as AST, ALT, GGT, SCr) were significantly different among groups. In addition, the QTc interval was significantly increased in the subjects with isolated IFG, isolated IGT, combined IFG and IGT, and type 2 diabetes compared with the subjects with normal glucose regulation (416.63 ± 24.79, 418.53 ± 24.20,419.2 ± 27.35, and 420.79 ± 25.49, respectively, vs. 415.6 ± 24.30, *p* < 0.05) (Fig. 1). The QTc interval was positively associated with abnormal glucose metabolism.

**Table 1:** Clinical and laboratory characteristics according to different glucose tolerance

Characteristics	DM	IFG + IGT	IGT	IFG	NGT	*P* value
N	1833	717	1255	773	3412	
Age (yr)	58.21 ± 7.20	56.86 ± 7.46	55.22 ± 7.60	55.56 ± 7.60	53.47 ± 7.79	< 0.001
Sex (M/F)	747/1086	253/464	307/948	316/457	866/2546	< 0.001
QTc	420.79 ± 25.49	419.20 ± 27.35	418.53 ± 24.20	416.63 ± 24.79	415.60 ± 24.30	< 0.001
BMI (kg/m^2^)	25.18 ± 3.38	25.06 ± 3.53	24.91 ± 10.43	24.38 ± 3.35	23.74 ± 3.25	< 0.001
WHR	0.90 ± 0.07	0.89 ± 0.09	0.88 ± 0.10	0.88 ± 0.08	0.86 ± 0.08	< 0.001
SBP (mm Hg)	136.51 ± 19.00	133.38 ± 17.22	127.74 ± 17.37	130.81 ± 18.01	123.80 ± 18.42	< 0.001
DBP (mm Hg)	81.78 ± 10.37	82.10 ± 9.51	79.97 ± 9.89	80.87 ± 9.66	77.79 ± 10.52	< 0.001
FPG (mmol/l)	8.05 ± 2.59	6.38 ± 0.24	5.57 ± 0.34	6.34 ± 0.22	5.41 ± 0.37	< 0.001
PPG (mmol/l)	13.41 ± 4.83	9.09 ± 0.89	8.92 ± 0.84	6.28 ± 1.02	6.08 ± 1.05	< 0.001
HbA1C (%)	6.91 ± 1.61	5.83 ± 0.41	5.74 ± 0.39	5.71 ± 0.41	5.60 ± 0.40	< 0.001
HbA1C (mmol/mol)	52 ± 17.6	44.67 ± 4.48	39.21 ± 4.26	38.89 ± 4.48	37.68 ± 4.37	< 0.001
Insulin(pmol/l)	8.92 ± 3.24	8.10 ± 3.89	7.27 ± 3.66	7.26 ± 3.35	6.27 ± 3.07	< 0.001
HOMA-IR	3.23 ± 0.94	2.36 ± 0.92	1.85 ± 0.93	2.11 ± 0.97	1.56 ± 0.78	< 0.001
HDL (mmol/L)	1.22 ± 0.32	1.23 ± 0.33	1.22 ± 0.28	1.28 ± 0.33	1.25 ± 0.32	< 0.001
LDL (mmol/L)	2.70 ± 0.79	2.71 ± 0.73	2.60 ± 0.73	2.72 ± 0.76	2.51 ± 0.74	< 0.001
CHO (mmol/L)	4.86 ± 1.08	4.85 ± 0.99	4.61 ± 0.99	4.84 ± 1.02	4.45 ± 0.98	< 0.001
TG (mmol/L)	2.08 ± 1.71	2.02 ± 1.11	1.73 ± 0.89	1.70 ± 0.91	1.38 ± 0.71	< 0.001
ALT (U/L)	20.86 ± 10.07	19.04 ± 10.31	16.99 ± 8.80	17.28 ± 8.04	13.97 ± 7.55	< 0.001
AST (U/L)	22.80 ± 10.95	21.88 ± 10.16	20.57 ± 9.22	21.36 ± 8.76	18.89 ± 9.03	< 0.001
GGT (U/L)	38.96 ± 14.55	33.49 ± 32.77	28.55 ± 36.90	31.04 ± 14.14	22.05 ± 11.11	< 0.001
Scr (umol/L)	68.71 ± 17.48	68.07 ± 19.95	64.03 ± 12.94	67.91 ± 14.05	63.34 ± 12.06	< 0.001
Smoking (n/total)	492/3412	161/773	156/1255	115/717	359/1833	< 0.001
Drinking (n/total)	705/3412	222/773	227/1255	179/717	449/1833	< 0.001

Values are mean ± SD or median (interquartile range) or number (proportion)

*p* values were for the ANOVA or χ^2^ analyses across the five groups

### Association between QTc interval and clinical characteristics

Table 2 presents the results of correlation analyses of the QTc interval with metabolism-related parameters. QTc levels correlated positively with age, sex, WHR, SBP, DBP, PPG, FPG, HbA1c, insulin, HOMA-IR, LDL, CHO, TG, smoking and drinking. After adjusting for age and sex, partial correlation coefficient values indicated that WHR, SBP, DBP, PPG, FPG, HbA1c, insulin, HOMA-IR, CHO and TG were still positively associated with the QTc interval. However, neither LDL (r = 0.021; *P* = 0.28), smoking (r =  − 0.002; *P* = 0.12) or drinking (r =  − 0.007; *P* = 0.39) correlated with the QTc interval.

**Table 2:** Crude and partial correlation between QTc and clinical parameters in the studied subjects

Variable	Crude r	Partial r^†^
Age (year)	0.139**	–
Sex	0.148**	–
BMI (kg/m^2^)	0.01	0.006
WHR	0.031*	0.039*
SBP (mmHg)	0.094**	0.085**
DBP (mmHg)	0.054**	0.081**
PPG (mmol/L)	0.091**	0.075**
FPG (mmol/L)	0.084**	0.083**
HbA1C (mmol/mol)	0.074**	0.053**
Insulin (pmol/L)	0.061**	0.045**
HOMA_IR	0.068**	0.054**
HDL (mmol/L)	0.014	0.004
LDL (mmol/L)	0.046**	0.021
CHO (mmol/L)	0.068**	0.041**
TG (mmol/L)	0.062**	0.071**
Smoking	− 0.106**	− 0.002
Drinking	− 0.083**	− 0.007

*p < 0.01; **p < 0.001

^†^Adjusted for age, sex

### Determinants of QTc interval

A multiple regression analysis with a stepwise model was used to assess the independent variables that may affect QTc levels. The variables entered in the model were as follows: age, sex, FPG, PPG, HbA1c, SBP, DBP, CHOL, TG, WHR, insulin, BMI, smoking, and drinking. The main determinants of QTc levels were age (β = 0.125, *p* < 0.001), sex (β = 0.192, *p* < 0.001), FPG (β = 0.065, *p* < 0.001), PPG (β = 0.052, *p* < 0.001), HbA1c (β = 0.043, *p* < 0.001), SBP (β = 0.044, *p* 0.003), DBP (β = 2.498, *p* 0.012), and TG (β = 0.049, *p* < 0.001) (Table 3).

**Table 3:** Multiple stepwise regression analysis showing variables independently associated with QTc

Independent variables	Standardized β	t	*P* value
Age	0.125	10.615	< 0.001
Sex	0.192	17.004	< 0.001
FPG	0.065	5.706	< 0.001
PPG	0.052	4.557	< 0.001
HbA1c	0.043	3.819	< 0.001
SBP	0.044	3.007	0.003
DBP	0.036	2.498	0.012
TG	0.049	4.358	< 0.001

The analysis also included CHOL, WHR, Insulin, BMI, smoking, and drinking which were all excluded from the model

### Associations between different glucose tolerance and QTc intervals

To further explore the relationship between different glucose tolerance and QTc intervals, multiple regression analyses were performed. The analyses revealed that isolated IGT (β = 0.044, *p* 0.002), combined IFG/IGT (β = 0.032, *p* 0.023) and type 2 diabetes (β = 0.076, *p* < 0.001) were significantly associated with the QTc interval after adjusting for all potential confounders (age, sex, insulin, SBP, DBP, CHOL, TG, smoking, and drinking) (Table 4).

**Table 4:** Adjusted associations with QTc according to different glucose tolerance

	Glucose tolerance categories	Standardized β	t	*P* value
Model 1	NGT	Reference		
	IFG	0.012	1.042	0.298
	IGT	0.043	3.561	< 0.001
	IFG + IGT	0.041	3.522	< 0.001
	DM	0.087	7.196	< 0.001
Model 2	NGT	Reference		
	IFG	0.027	1.938	0.053
	IGT	0.044	3.043	0.002
	IFG + IGT	0.032	2.279	0.023
	DM	0.076	5.049	< 0.001

Model 1 not adjusted

Model 2 adjusted for age, sex, insulin, SBP, DBP, CHOL, TG, smoking, and drinking

### Associations between different glucose tolerance and QTc prolongation

In the present study, we considered QTc > 440 ms as abnormally prolonged. Taking NGR as a reference, isolated IGT, combined IFG and IGT, and type 2 diabetes were all risk factors for prolonged QTc.The OR of IGT for QTc prolongation was 1.131 (95% CI 1.059–1.356), the OR of IFG + IGT for QTc prolongation was 1.396 (95% CI 1.126–1.73), and the OR of T2DM for QTc prolongation was 1.34 (95% CI 1.142–1.577) (*P* for trend < 0.001) after adjusting for age, sex, insulin, SBP, DBP, CHOL, TG, smoking, and drinking (Table 5).

**Table 5:** Adjusted ORs and 95% CIs for prolonged QTc according to different glucose tolerance

Glucose tolerance categories	n/total	Crude OR (95% CI)	Adjusted OR (95% CI)
NGT	483/3412	1	1
IFG	121/773	1.129 (0.910–1.402)	1.110 (0.888–1.387)
IGT	216/1255	1.264* (1.061–1.506)	1.131* (1.059–1.356)
IFG + IGT	144/717	1.538** (1.251–1.890)	1.396** (1.126–1.730)
DM	361/1833	1.486** (1.279–1.727)	1.342** (1.142–1.577)

Adjusted OR: adjusted for age, sex, lipid profiles, BMI, SBP, and DBP, smoking, and drinking

*p < 0.01; **p < 0.001

## Discussion

The main finding of this study is the effect of different glucose tolerance on the QTc interval. QTc interval prolongation is more common in patients with isolated IGT, combined IFG and IGT, and type 2 diabetes than in participants with NGR. Moreover, this association is independent of potentially confounding covariates.

Cardiac autonomic dysfunction present in prediabetes may lead to repolarization disturbances and may increase the risk of ventricular arrhythmias and sudden cardiac death [21]. Many ECG parameters, such as QTc prolongation, enhanced QT dispersion, the short-term variability of the QT interval, p-wave dispersion and heart rate variability were verified in patients with diabetes and IGT [13, 21–23]. ECG abnormalities were independently associated with an increased risk of developing coronary heart disease in a population of Middle Eastern women [24]. QTc prolongation is reported to be an independent risk factor for coronary heart disease in type 1 and type 2 diabetes and has been described to be a prominent predictor of cardiac death even in newly diagnosed type 2 diabetes patients [25, 26]. Many studies have demonstrated that QTc prolongation has direct detrimental effects on deadly arrhythmias and the risk of sudden death with myocardial ischemia [6, 13]. Additionally, hypoglycemia is associated with a significant prolongation of the QTc interval [27]. The above studies support the role of the QTc interval in predicting the risks of mortality. In this study, we found that the QTc interval was prolonged in prediabetes and diabetes which indicates that people with IGR and diabetes may be at high risk of arrhythmia and cardiovascular disease (CVD) mortality.

Previously published articles have reported that age [13], sex [5], BMI [28], hypertension [29], insulin concentration and hyperglycemia [30] are all risk factors for prolonged QTc interval in patients with diabetes. Consistent with the above studies, our analysis showed that age, sex, SBP, DBP, PPG, FPG, HbA1c, TG were positively correlated with the QTc interval. However, after further adjusting for the related confounding variables, BMI, insulin, HOMA-IR, HDL, LDL and CHO were no longer independently related to the QTc interval. In agreement with our results, the EURODIAB Prospective Complications Study demonstrated that female sex and higher values of HbA1c and systolic blood pressure were associated with a higher incidence of prolonged QTc, whereas BMI and physical activity within the range of 21.5–23.2 kg/m^2^ were displayed as protective factors [31].

In the present study, we further evaluated the association between different glucose tolerance and prolonged QTc intervals using a logistic regression model. Our results agreed with previous findings that patients with IGR, a prediabetic condition, were correlated with QTc interval [21, 31]. However, surprisingly, impaired fasting glucose was not found to significantly influence the QTc interval, which is not in agreement with other studies [32, 33]. The reason for this is probably because the participants in our study were from only one area and more women were recruited than men.

Hyperglycemia may cause QTc prolongation by several mechanisms including the stimulation of protein kinase C, which can reduce the synthesis and release of endothelial derived nitric oxide [34], consequently resulting in a decrease in Na-K-ATPase activity. The reduced nitric oxide bioavailability during hyperglycemia may also be responsible for the decreased activity of Ca^2+^ ATPase [35], an enzyme that maintains a low concentration of Ca^2+^ ions in the cell. Decreased activity of Na-K-ATPase will increase the intracellular calcium concentration and extend the QTc interval. Moreover, hyperglycemia is related to increased sympathetic activity as shown by increased plasma catecholamine concentrations [36]. Sympathetic stimulation unopposed by vagal activity may also induce ventricular electrical instability.

In summary, our study shows that prediabetes and diabetes appear to be independent risk factors for QTc interval prolongation after controlling for potential cofounders.The QTc interval is remarkably associated with glucose metabolism indices (including FPS, PPS, and HbA1c). In view of this, it is reasonable to consider whether the QTc interval alone should become a novel target for clinical intervention. Our study supports the concept that postprandial glucose level elevation may have an important role in the pathogenesis of cardiovascular complications [31]. Consistent with the previous study [37], we found that hyperglycemia, especially postprandial is an important risk factor for QTc prolongation in this study. Impaired glucose regulation appears to be an indication to evaluate and monitor the QTc interval in the population. We believe that our data are relevant in terms of public health. Further research is needed to verify whether glucose control among people with impaired glucose regulation may be useful in reducing QTc interval prolongation and ultimately limiting the risk of ventricular arrhythmia and sudden death.

The advantage of the present study is that we evaluated the relationship between all different glycometabolism conditions and the QTc interval in a large-scale population. Our study also has some limitations. First, given the cross-sectional nature of the study design, a cause-effect relationship between abnormal glucose metabolism and the QTc interval could not be verified. Further prospective studies are needed to analyze the potential pathophysiological mechanism of this correlation. Second, inaddition to the QTc interval, other markers of cardiac repolarization abnormalities, such as QT dispersion, were not evaluated. Third, because our data were obtained from middle-aged and older subjects, and the gender proportions were unbalanced, it has yet to be seen whether our results can be generalized to younger populations or other ethnic groups. Last, the QTc intervals were from one ECG which might not represent the true resting heart rate.

## Data Availability

The data that support the findings of this study are available from the corresponding author on reasonable request. Inquiries for data access may be sent to the following e-mail address: suqing@xinhuamed.com.cn.

## Abbreviations

QTc: Corrected QT
IGR: Impaired glucose regulation
IFG: Impaired fasting glucose
IGT: Impaired glucose tolerance
DM: Diabetes mellitus
WHR: Waist-to-hip ratio
BMI: Body mass index
SBP: Systolic blood pressure
DBP: Diastolic blood pressure
OGTT: Oral glucose tolerance test
FPG: Fasting plasma glucose
2 h PPG: 2-H postload plasma glucose
HbA1c: Hemoglobin A1c
TC: Total cholesterol
TG: Triglycerides
LDL: Low-density lipoprotein-cholesterol
HDL: High-density lipoprotein-cholesterol
ALT: Alanine aminotransferase
AST: Aspartate aminotransferase
SCr: Serum creatinine
